# Temporal changes in avian community composition in lowland conifer habitats at the southern edge of the boreal zone in the Adirondack Park, NY

**DOI:** 10.1371/journal.pone.0220927

**Published:** 2019-08-19

**Authors:** Michale J. Glennon, Stephen F. Langdon, Madeleine A. Rubenstein, Molly S. Cross

**Affiliations:** 1 Wildlife Conservation Society, Saranac Lake, NY, United States of America; 2 Shingle Shanty Preserve and Research Station, Long Lake, NY, United States of America; 3 National Climate Adaptation Science Center, U.S. Geological Survey, Reston, VA, United States of America; 4 Wildlife Conservation Society, Bozeman, MT, United States of America; University of Waikato, NEW ZEALAND

## Abstract

Climate change represents one of the most significant threats to human and wildlife communities on the planet. Populations at range margins or transitions between biomes can be particularly instructive for observing changes in biological communities that may be driven by climate change. Avian communities in lowland boreal habitats in the Adirondack Park, located at the North American boreal-temperate ecotone, have been the focus of long-term monitoring efforts since 2007. By documenting long-term changes in community structure and composition, such datasets provide an opportunity to understand how boreal species are responding differently to climate change, and which habitat characteristics may be best able to retain boreal avian communities. We examined three specific questions in order to address how well current biological communities in Adirondack boreal wetland habitats are being maintained in a changing climate: (1) how do trends in occupancy vary across species, and what guilds or characteristics are associated with increasing or decreasing occupancy? (2) how is avian community composition changing differently across sites, and (3) what distinguishes sites which are retaining boreal birds to a higher degree than other sites? Our analysis revealed that (1) boreal species appear to exhibit the largest changes in occupancy among our study locations as compared to the larger avian community, (2) dynamics of community change are not uniform across sites and habitat structure may play an important role in driving observed changes, and (3) the particular characteristics of large open peatlands may allow them to serve as refugia for boreal species in the context of climate change.

## Introduction

Climate change is among the most significant threats to ecological communities worldwide and can lead to profound changes to the structure of biological communities [1]. Documented or predicted patterns of shifting community structure resulting from climate change include avian turnover rates of 20–40% [2,3], shifts toward increased representation of resident birds and southern species [4,5,6], declines in habitat specialists and cold-adapted species [5], increases in species richness and mean body mass among winter bird communities [7], and shifting competitive relationships among community members [8].

Populations at range margins or transitions between biomes can be particularly instructive for observing changes in biological communities that may be driven by climate change [9,10]. The Adirondack Park in New York State lies at the North American boreal-temperate ecotone [11] and is in the southern edge of the range for several species of birds whose breeding distribution is primarily within the boreal zone of eastern North America. Avian communities in lowland boreal habitats in the Adirondack Park have been the focus of long-term monitoring efforts since 2007, with a particular focus on 8 boreal species including black-backed woodpecker (*Picoides dorsalis*), boreal chickadee (*Poecile hudsonicus*), Canada jay (*Perisoreus canadensis*), Lincoln’s sparrow (*Melospiza lincolnii*), palm warbler (*Setophaga palmarum*), olive-sided flycatcher (*Contopus cooperi*), rusty blackbird (*Euphagus carolinus*), and yellow-bellied flycatcher (*Empidonax flaviventris*). The habitats of these boreal specialists are thought to be particularly vulnerable to climate change [12,13] and declining occupancy patterns have been documented for a number of these species [14,15].

Lowland boreal habitats in the Adirondack Park consist of open peatlands, conifer dominated forested peatlands, and neighboring conifer dominated upland communities. These habitats are patchily distributed and landscape characteristics such as size, connectedness, structure, and anthropogenic disturbance to wetland habitats appear to interact with climate to influence dynamics of birds in these locations. Because they tend to be cooler than surrounding landscapes [16], peatlands may provide non-peatland taxa with temporary refugia as they retreat to higher latitudes and altitudes. Peatlands are often isolated within anthropogenically modified landscapes and are some of the last wild places; they therefore can provide stepping stones for the migration of more adaptable species that are unable to survive in agricultural or other strongly modified landscapes [17].

We have found that boreal habitat specialists in these communities are in decline and appear sensitive to changing climate [14]. Boreal wetlands also function as habitat for other species of birds including those which may expand into these habitats as more southerly species shift their ranges north in a changing climate. We examined the dynamics of the broader avian community in boreal wetlands in order to explore how these communities may be changing with changing temperature and precipitation patterns. Poleward range shifts associated with climate change have been documented on all continents and in most major oceans [1]. Boreal species, on their southern range extent in the Adirondacks, may move northward and generally be replaced by more southern species. Similarly, climate change has been linked to changes in arrival dates [18]. Changes to habitat structure resulting from climate or other mechanisms may drive changes in primary habitat and nesting guilds, while wintering ground habitat changes may result in observed changes among guilds of birds wintering in variable geographies. Our dataset encompasses all passerines and woodpeckers, as well as our target species, and provides, therefore, an opportunity to examine community level changes in peatland bird communities. We tested a broad range of species characteristics in order to explore community structural changes and their potential relationship to species’ use of habitat, distribution and abundance, and/or life history characteristics.

Climate change does not operate uniformly across landscapes. There is high variability in temperature and precipitation characteristics across our study site locations in the Adirondack Park, NY and some sites may be better suited to harbor species over the long term than others. Climate refugia are defined as areas relatively buffered from contemporary climate change over time such that important physical, ecological, and social characteristics are able to persist [19]. One possible means of identifying climate refugia is to identify locations where current temperature and precipitation patterns have most closely tracked historical patterns [20]. Another may be to identify locations characterized by relatively stable biological communities. Here we describe changes in avian communities inhabiting boreal peatlands in the Adirondacks from 2007–2016 and identify characteristics of species and sites that are exhibiting the greatest and lowest degree of change. Our purpose, in particular, is to identify characteristics associated with sites with high and low degrees of change in the boreal component of their bird fauna, and the degree to which decline among the boreal group may be associated with changes in other species and with site characteristics. As northern birds disappear from these habitats, understanding who may be replacing them, and where changes are occurring at high or low rates can help to identify potential refugia and to point toward strategies for long-term protection of boreal habitats. Specifically, we address the following questions (1) how do trends in occupancy vary across species, and what guilds or characteristics are associated with increasing or decreasing occupancy? (2) how is avian community composition changing differently across sites, and (3) what distinguishes sites which are retaining boreal birds to a higher degree than other sites and therefore may be refugia for these species?

## Methods

### Study site locations

Our study occurred in the Adirondack Park, an area of 19,700 km^2^ located in the northern part of New York State in the US (43°58’14” N, 74°03’12” W). The predominant habitat type in the park is Northern Hardwood and Conifer Forest, followed by Boreal Upland Forest and Northern Swamp [21,22]. Though the Adirondacks as a whole lie in the transition zone between the temperate and boreal regions, there are extensive areas in the park that are characterized by boreal community types, with summer temperatures characteristic of the southern edge of the true boreal, and maintained by boreal processes such as ice buildup on river shores [14,12]. The boreal habitats that are the subject of this study consist of bogs, fens, wooded wetlands, and open river corridors in the Adirondack Park and have been described previously [14]. Boreal habitats of the Adirondacks are found in montane and lowland ecosystems. Montane boreal habitats are the focus of a separate high-elevation bird monitoring program in this region [23]. This study focuses on lowland boreal ecosystems that are generally restricted to large peatland complexes. Such habitats include open and forested bogs, fens, low-gradient riparian corridors and (less-frequently) conifer dominated glacial outwash plains. As recently characterized [22], boreal communities in the Adirondacks fall primarily into Northern Swamp, Northern Peatland, and Boreal Upland Forest macrogroups, with dominant habitat types within those macrogroups including Northern Appalachian Acadian Conifer Hardwood Acid Swamp, Boreal Laurentian-Acadian Acidic Basin Fen, Boreal Laurentian Bog, and Acadian Low Elevation Spruce Fir Forests and Sub-Boreal Spruce Flats. These are predominantly wet, acid, carbon-accumulating habitats with mean summer temperature < 18°C and predominantly coniferous vegetation. Dominant vegetation includes conifer trees such as black spruce (*Picea mariana*) and tamarack (*Larix laricina*), ericaceous shrubs such as leatherleaf (*Chamaedaphne calyculata*) and Labrador tea (*Ledum groenlandicum*), herbaceous plants such as *Sarracenia purpurea* (pitcher plant) and sedges (e.g., *Carex* spp., *Eriophorum* spp.), and *Sphagnum* mosses.

As described in [14], an initial list of potential field sites was compiled by consulting a variety of data sources including Adirondack Park Agency wetlands inventory data, New York State Breeding Bird Atlas data [24,25], postings to the Northern New York Breeding Bird Listserv, and local expert opinion. The final list of study sites was then determined by selecting from within the potential list to include a number of the major well-known boreal wetlands of the Adirondack Park and a random sample of smaller, lesser-known locations.

### Sampling

We conducted unlimited distance point counts to assess presence/absence of passerines and woodpeckers along transects of 5 points spaced at least 250m apart within boreal wetland habitats [26]. We employed spatial replication of sample points rather than temporal, to reduce travel costs. Both spatial and temporal replication allow for the calculation of detection probabilities [27,14]. The sites themselves, and not the five points within each site, serve as the units for the purposes of analysis. All points were surveyed for 10-minutes between the hours of 5:00 and 9:00 am during the primary breeding season on survey dates ranging from the last week of May to the third week of July, with the majority of sites sampled in June. Surveys were conducted by trained observers, the majority of whom conducted counts for 3 or more of the project years. During counts, we recorded the date, start and end time for each survey, ambient temperature, and sky and wind conditions. We have sampled more than 80 locations over the course of the study; a total of 58 sites have been sampled regularly from 2007–2016. Data from these sites form the basis of the analysis described here. Our non-invasive sample method required no specific research permits. Nearly all sites were located on public land; permission for access was obtained on the small number of private land sites.

### Question 1: Individual species change

To identify the characteristics of individual species whose occupancy is changing more or less rapidly in boreal habitats (Question 1), we first used the multi-season model implemented in program Presence [28] to calculate detection (p), occupancy (ψ), colonization (γ), and extinction (ε) probabilities for 2007–2016 for each of the species for which adequate data were obtained (detections at 15% or more of study locations; [29]. We modeled detection for each species and tested 6 variables for their influence on detection probability including wind, sky (relative cloud cover), date, time, temperature, and observer (as per [14]). We used the default parameterization of the multi-season model, which estimates initial occupancy, colonization, and extinction probabilities directly, and is the most numerically stable [27]. We estimated trends in occupancy over time across all sites without attempting to model specific influences on the vital rates of extinction and colonization in this part of the analysis. The only covariates included here were those influencing detection. We used information from top models (ΔAIC ≤ 2.0) for each species to estimate occupancy in year 1, as well as colonization and extinction rates. Occupancy rates for years 2–10 were calculated from initial ψ, γ, and ε. We calculated growth rate (λ) as ψ_t+1_/ψ_t_ and obtained a rate of change in occupancy for the study period by calculating the geometric mean of individual growth rates (λ_t_) between all years [30]. A total of 71 species were detected in at least 15% of study locations but individual species models failed for 13 species, resulting in a total of 57 for which occupancy trends were obtained.

In order to identify potential mechanisms underlying individual species’ changes, we used analysis of variance and regression to examine a set of species characteristics for their association with trend parameters. We tested 9 categorical and 5 continuous species characteristics including (1) boreal, (2) southern, (3) feeding guild, (4) foraging guild, (5) nesting location, (6) primary habitat, (7) migratory strategy, (8) winter geography, (9) single- or multiple-brooded, (10) mean clutch size, (11) mean body weight, (12) mean latitude of New York State distribution, (13) estimated NY population, and (14) mean arrival date (S1 File).

Both “boreal” and “southern” are categorical descriptions of species distributions. Boreal species are those which have been the specific targets of long-term monitoring in boreal habitats in the Adirondacks [14] and are defined by having a primarily Canadian boreal distribution, a location at or near their southern range extent in our study area, and a distribution within the Adirondack Park that is highly associated with lowland boreal habitat types. Other species of passerines have similar northern distributions (e.g., Swainson’s thrush (*Catharus ustulatus*), blackpoll warbler (*Setophaga striata*)) but are more commonly associated with high elevation conifer communities and uncommon in the habitats sampled here.

Southern species were those defined as having a latitudinal center of their distribution that is south of the southern boundary of the Adirondack Park. We used the mean latitude and longitude of Breeding Bird Atlas blocks in which each species occurred within New York State during the most recent atlas [25] to identify those species whose distributions in NY are centered to the south of the Adirondacks.

Information related to feeding (insectivore, omnivore), foraging (bark gleaner, flycatcher, foliage gleaner, ground forager), nesting (cavity, ground, shrub, tree), primary habitat (human commensal/generalist, conifer forest, forest interior, forest generalist, open land, shrub/marsh), migratory strategy (nonmigratory, short-distance, long-distance), winter geography (Caribbean, Central/South America, Resident, US/Canada, Widespread), number of broods (single, multiple), clutch size, and body weight was obtained from a variety of sources [31,32,33,34]. Open land birds in the context of this study refers to those of both open woodland habitats and some types of agricultural landscapes with grass and scattered trees such as farmlands and orchards, rather than to birds of open peatlands specifically, which are captured in the boreal guild. Mean latitude of distribution in New York was obtained in the same manner as that which determined southern species, but was numerical rather than categorical. Estimated New York population was obtained from the Partners in Flight population estimates database [35]. Mean arrival dates for our study period were obtained from eBird and depicted the Julian date of the first day of the week during which each species was first observed in Franklin County. We chose Franklin County in order to separate resident birds from birds who are located in NY year round, but not in our study sites in the northern Adirondacks. We used ANOVA (categorical) and regression (continuous) in Systat 12.0 (Systat Software, San Jose, CA) to determine the variability in growth rates (λ) that could be explained by these characteristics.

The questions of concern in the individual species analysis were: (1) which species are exhibiting patterns of increasing or declining occupancy in boreal wetlands (2) are there commonalities among species exhibiting particular patterns of change, i.e., to what degree to guild types explain changes among individual species?

### Question 2: Site-level community change

The purpose of the second part of our analysis was to examine the process of avian community change individually at each study site and to determine whether observed patterns of change differ among these sites. In contrast to the first part of our analysis in which we examined individual species occupancy patterns and associated guilds/characteristics, here we model each site independently and examine changes in species richness, such that it is possible for processes of avian community structural change to vary across sites.

MacKenzie et al. [27] describe an approach to community analysis wherein one may examine the community at one location and treat each species as an analog of a “site,” and in which guild or other species characteristics can be modeled as “site covariates” in a manner similar to more standardized occupancy modeling methods. In this alternative approach, the occupancy parameter (ψ) is analogous to relative species richness [36] and is the proportion of the total species pool present at the site. With respect to our study, the total species pool is defined as the list of species that have been detected at our sites at least once [27] and is 71. By placing covariates such as guild assignments on occupancy or dynamic rates, individual estimates of guild occupancy and change in guild occupancy can be obtained that are analogous to relative species richness and change in relative species richness among guilds. Because each site is modeled separately, the advantage of this approach over other methods of community structural analysis (e.g., simultaneous modeling of species, [27]) is that it does not constrain the pattern of changes across sites (e.g., a particular guild may be increasing in some locations and decreasing in others) and it allows for the explicit examination of changes in species richness which may result from climate or other landscape changes (e.g., increases in richness of southern birds as a result of poleward range shifts).

We took this approach to investigate characteristics of change among bird guilds. For each of our individual study locations, we ran two model sets to examine increases (colonization) and decreases (extinction) in relative species richness for the decadal period of 2007–2016, and used covariates to determine how changes in relative species richness were driven by particular species characteristics (as described previously under Question 1). Specifically, for each individual location, we accounted for detection and, using the same set of factors explored above (e.g., boreal, southern, foraging, etc.), restricted each model to a single covariate placed on either colonization or extinction (S1 Table). With complete model sets for each study location, we extracted colonization and extinction rates for all species characteristics and examined top models to determine which factors were most strongly associated with dynamic rates. The primary question of interest in this part of our analysis was to determine whether or not the process of community change differs across our study sites and, if so, why.

### Question 3: Boreal decline

The purpose of the final part of our analysis was to determine what distinguishes those locations which are retaining boreal birds to a higher degree than others and therefore may be refugia for these species. The primary aim of our long-term monitoring has been to identify trends and drivers of change for a suite of boreal birds in Adirondack peatlands, a group for which declining patterns of occupancy have been documented in 6 of 8 tracked species [15]. As such, in the final part of our analysis we focused on rates of change in relative species richness of the boreal guild, and used analysis of variance to identify characteristics of sites with varying degrees of loss within the boreal bird community. Extinction rates for the boreal guild were extracted from models for each individual site and we used cluster analysis (K-means, Systat 12.0) to place study locations into 3 groups: sites were categorized as having either high, medium, or low boreal bird extinction rates. We then examined 3 factors we hypothesized might be associated with loss of boreal bird species richness–(1) changes in relative species richness among other, potentially competitive guilds, (2) habitat type, and (3) climatic stability. We specifically tested whether colonization rates of commensal and/or southern species differed among locations with low, medium, and high levels of loss in the boreal bird guild. With respect to habitat, we tested whether the proportion of Boreal Upland Forest, Northern Peatland, and Northern Swamp at each study location differed among areas of low, medium, and high extinction rates. Habitats were obtained from the Northeast Terrestrial Habitat Classification Map (macrogroup level; [37]) and summarized within 500m of each study transect. Finally, to examine whether particular sites may be operating as climate refugia, we tested whether sites with high, medium, and low boreal extinction rates differed in terms of their deviation from long-term conditions. To do so, we used data from the Parameter-elevation Relationships on Independent Slopes model (PRISM [38]) at 800m resolution and obtained mean temperature and precipitation values for study site locations for all months and years between December 2006 and August 2016 as well as temperature and precipitation normal values, which represent average monthly and annual conditions over the most recent three full decades. These were used to calculate deviation in mean winter (December–March) and breeding (May–August) season temperature and precipitation from 30-year normals for each study location. The essential questions with which we were concerned in this final part of our analysis were: (1) how does loss of the boreal bird guild vary among sites, and (2) what factors distinguish sites with varying degrees of loss?

## Results

### Question 1: Individual species change

Our initial aim was to examine individual species trends across sites and to identify the characteristics of individual species whose occupancy is changing more or less rapidly in boreal habitats. Across all species (57 for which trends could be calculated), we identified 35 species which had λ > 1 and 22 with λ < 1 (S1 File). A growth rate < 1 indicates a pattern of declining occupancy, whereas λ > 1 indicates an increase in occupancy probability [39]. For 44 species (63%), occupancy change (positive or negative) indicated by λ was less than 5% annually. With respect to species characteristics, only the boreal and foraging guilds had any explanatory power to distinguish rates of change among species. Boreal species had lower growth rates (mean = 0.955) than other species (mean = 1.017; F = 7.925, P < 0.007) and flycatchers had lower growth rates (mean = 0.973) than bark foragers (mean = 1.064), foliage gleaners (mean = 1.003), and ground foragers (mean = 1.004; F = 2.853, P < 0.046). Additional, non-categorical species characteristics (i.e., clutch size, weight, mean latitude of NY distribution, NY population, arrival date) were not significantly related to rates of occupancy change.

**Fig 1:**
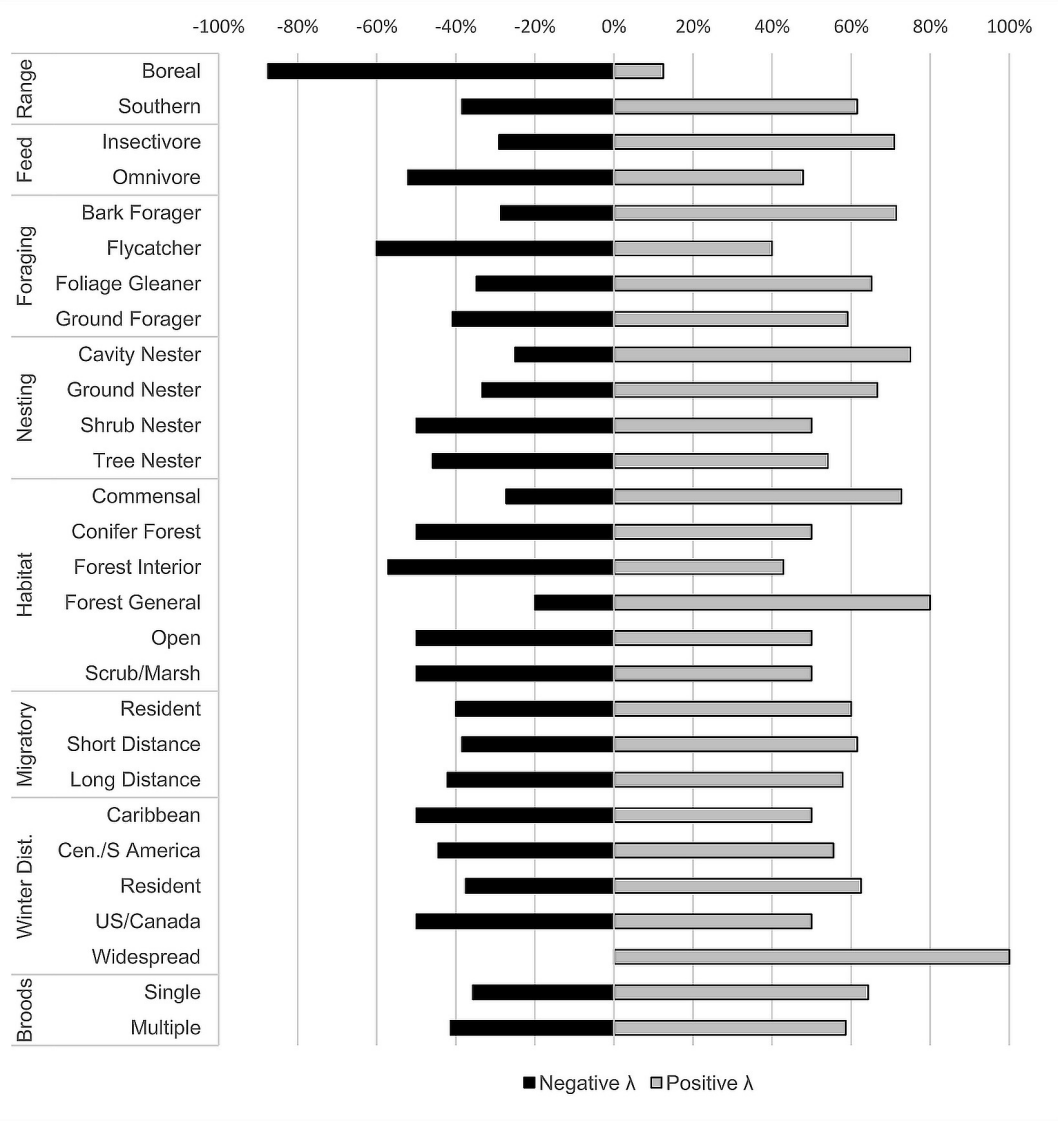
Proportion of bird species within each group with positive and negative change in occupancy (λ).

Though no other species characteristics were significantly related to growth rates, patterns of increase and decrease indicate that, in addition to boreal species and flycatchers, several guilds show a roughly equal split between number of species with positive λ and those with negative λ (e.g., shrub nesters, open land species; Fig 1), indicating that guild alone was not a useful predictor of changes in occupancy probability. Groups that appear to be increasing for the most part include insectivores, bark foragers, cavity nesters, commensal/generalist species, forest breeding birds, and species whose wintering distribution is widespread (occurring in roughly equal abundance in 3 or more geographic regions [40]). Guilds are, of course, not mutually exclusive and so patterns of decline or increase may be the result of shared species among a number of guilds. With respect to individual species, the boreal group was among those with lowest λ, but additional species with λ < 1 in our study site locations included several more common species such as scarlet tanager (*Piranga olivacea*), American goldfinch (*Spinus tristis*), and least flycatcher (*Empidonax minimus*). Among those with highest λ were pine warbler (*Setophaga pinus*), pileated woodpecker (*Drycopus pileatus*), Eastern bluebird (*Sialia sialis*), and Swainson’s thrush (S1 File).

### Question 2: Site-level community change

**Fig 2:**
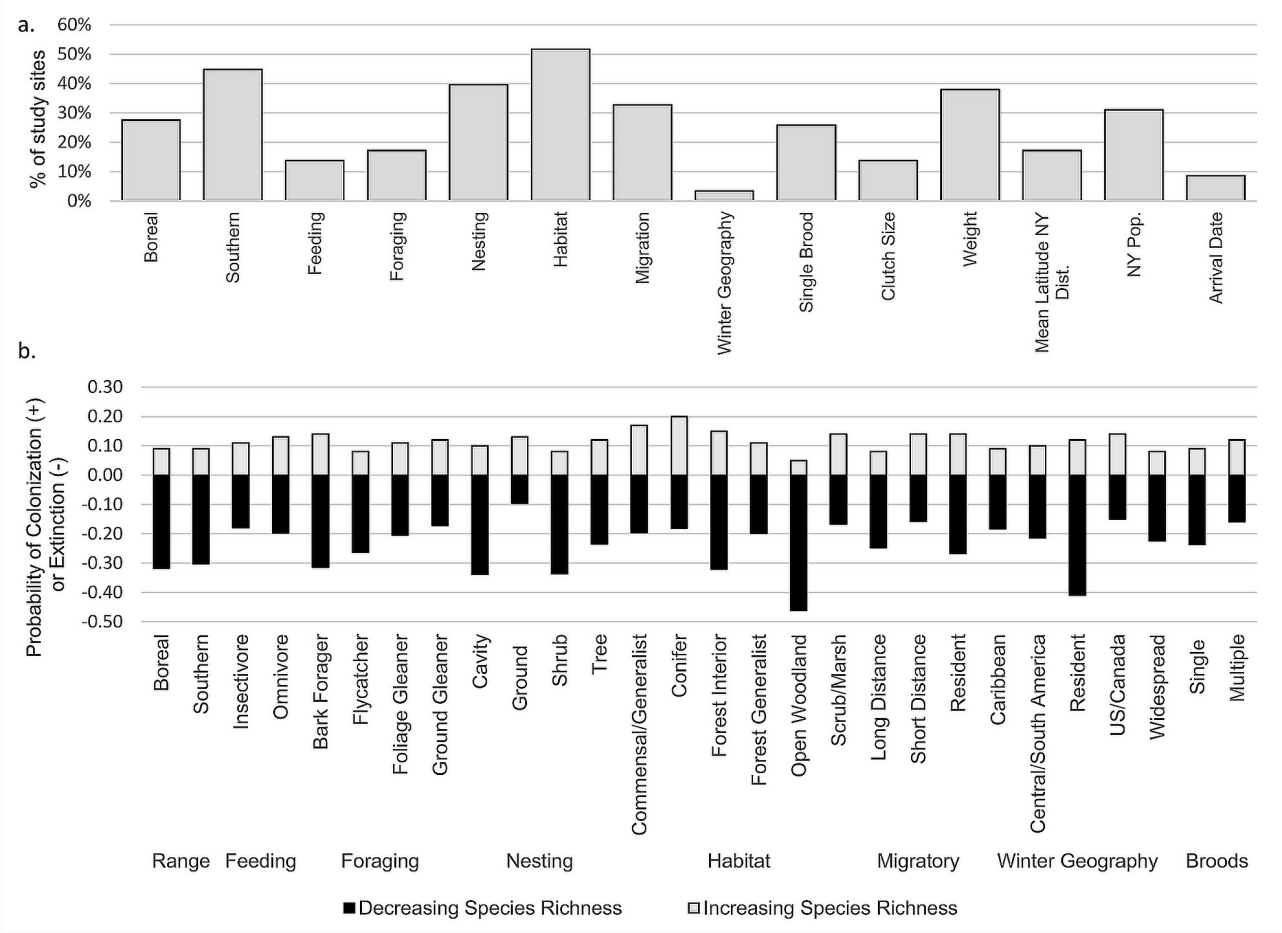
Influence of species characteristics on the process of community change in boreal habitats. (a) Proportion of study sites in which species characteristics occurred in top models (AIC < 2.0) of gain (colonization) or loss (extinction) of species richness. (b) Mean rates of increase (colonization) and decrease (extinction) in species richness among bird groups across study sites.

Our second question was, do observed patterns of avian community change differ among sites? We used data from each study location to run two model sets to examine increases (colonization) and decreases (extinction) in relative species richness for 2007–2016, and used covariates to determine how changes in relative species richness were driven by species characteristics. Combining information from results of model selection at all sites led to a number of general patterns. We found that (1) the process of community change among these habitats is highly variable, with no one factor most critical in driving increases or decreases in species richness across all sites, and (2) for most groups, loss of species richness appears more likely than gain (Fig 2, S2 Table).

Several characteristics appear to be important to driving patterns of increasing or decreasing species richness among our study locations (Fig 2A). Across sites, habitat was included most often in top models for colonization or extinction, followed by southern distribution and nesting guild. Colonization rates (or increases in relative species richness) were highest for conifer species, followed by commensal/generalists, and lowest for open land birds; by nesting guild, colonization was higher for ground and tree nesting species than for cavity and shrub nesters (Fig 2B).

With respect to extinction, rates were highest for open land birds and lowest for conifer forest species, and, by nest location, extinction was highest for shrub nesting birds, followed by cavity, tree, and ground nesters. Combining information from both dynamic rates, it appears that conifer species and commensal/generalists are among the most likely to colonize boreal wetland habitats and among least likely to leave them, while birds of open lands are the least likely to colonize and most likely to abandon these habitats. Though birds with distributions centered to the south of the Adirondacks were important in changing community structure within our study sites, they remain less likely to colonize these habitats than they are to leave them (Fig 2B). Among nearly all species groups, in fact, mean extinction rates were generally higher than colonization rates, suggesting that a loss of overall species richness may be occurring broadly across these habitats.

Noncategorical species characteristics such as clutch size also influenced colonization and extinction rates, but results for most continuous variables were mixed across our sites. Among these variables, mean body weight occurred most often in top models for colonization or extinction (n = 22 sites), followed by NY population (n = 18), latitude (n = 10), mean clutch size (n = 8), and arrival date (n = 5). In all cases, the influence of these characteristics on changes in species richness was mixed among sites, exerting a positive influence on colonization or extinction in some sites and a negative influence in others (S2 Table). The most consistent patterns indicated a generally positive effect of New York State population size on colonization rates (positive influence at 79% of sites), a positive effect of mean body weight on extinction rates (91% of sites), and a negative effect of arrival date on both colonization (86% of sites) and extinction rates (89% of sites).

### Question 3: Boreal decline

We utilized the third component of our analysis to attempt to discern factors associated with decreasing representation of the boreal guild, and to determine the characteristics of sites that may be serving as refugia for these species. The primary focus of long-term monitoring in these habitats [14] has been on the species that are most closely associated with them and these species generally appear to be a declining component of the avifauna. The mean colonization rate, or likelihood of increases in relative species richness of the boreal guild across all sites was 0.09, while the mean extinction rate across all sites was 0.33, indicating that boreal birds may be a diminishing component of these communities in most of our study wetlands, which is in line with individual species occupancy trends for this group.

**Fig 3:**
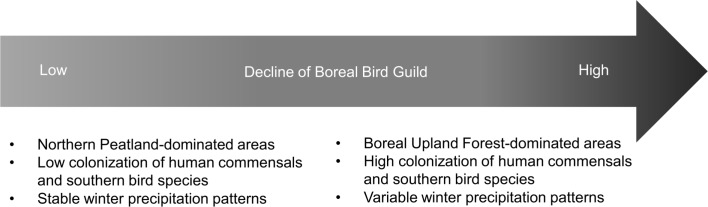
Characteristics associated with loss of boreal bird species richness in low elevation boreal wetlands.

We found that several factors were important in distinguishing sites characterized by high (mean = 0.73), medium (mean = 0.39), and low (mean = 0.11) levels of extinction (Table 1). We found that both southern species and commensal/generalist species had higher probability of colonization in sites with high boreal bird extinction rates. We also found that sites with lowest extinction rates for boreal birds were dominated by Northern Peatland habitat types and had lowest amounts of Boreal Upland Forest. Last, we found that climate stability in winter appeared to benefit boreal birds; sites with low boreal extinction rates were characterized by low deviation from normal winter precipitation. Low, medium, and high levels of boreal bird extinction were not distinguished by other climate characteristics or by the amount of Northern Swamp habitat at the study location. Overall, the loss of boreal bird species richness in these sites appears to be associated with increases in forest cover, colonization by southern and human commensal species, and variability in winter precipitation patterns (Fig 3).

**Table 1:** Factors used to distinguish sites with low, medium, and high extinction probability of boreal birds in low elevation boreal habitats in the Adirondack Park, NY, 2007–2016 (analysis of variance, superscripts denote the results of pairwise tests (P < 0.05), means with the same superscript do not differ).

Variable	Low	SE	Med	SE	High	SE	R^2^	F	P
Colonization Rate of Southern Species	0.08^a^	0.01	0.08^a^	0.01	0.13^b^	0.02	0.15	4.63	0.014
Colonization Rate of Commensal Species	0.15^a^	0.03	0.13^a^	0.03	0.28^b^	0.04	0.19	5.02	0.011
Boreal Upland Forest within 500m of Transect (%)	15^a^	0.04	34^b^	0.04	27^ab^	0.06	0.19	6.19	0.004
Northern Peatland within 500m of Transect (%)	34^a^	0.03	14^b^	0.04	16^b^	0.06	0.27	9.48	0.001
Northern Swamp within 500m of Transect (%)	23^a^	0.03	23^a^	0.03	22^a^	0.05	0.00	0.05	0.952
Deviation from Normal Winter Temperature (°C)	0.16^a^	0.02	0.22^b^	0.02	0.15^ab^	0.03	0.13	3.71	0.031
Deviation from Normal Breeding Season Temperature (°C)	0.34^a^	0.04	0.31^a^	0.04	0.35^a^	0.07	0.01	0.32	0.729
Deviation from Normal Winter Precipitation (cm)	5.70^a^	0.17	6.33^b^	0.17	6.45^b^	0.29	0.15	4.42	0.017
Deviation from Normal Breeding Season Precipitation (cm)	7.86^a^	0.35	7.75^a^	0.36	8.72^a^	0.60	0.04	1.00	0.374

## Discussion

The purpose of our work was to explore temporal changes in avian community composition in lowland conifer habitats with the aim of understanding the characteristics of both species and individual sites that are associated with high and low degrees of change, and to determine how these characteristics may help to identify refugia for boreal species. We asked, specifically, (1) how do trends in occupancy vary across species, and what guilds or characteristics are associated with increasing or decreasing occupancy (2) how is avian community composition changing differently across sites, and (3) what distinguishes sites which are retaining boreal birds to a higher degree than other sites.

### Question 1: Individual species change

We found, on the whole, a greater proportion of species increasing in occupancy over time than those that exhibited patterns of decline. Increases and decreases among individual species mimic those that have been found in other locations, but in general the only strong pattern observed was a loss of boreal bird species that have been the focus of long-term monitoring. Work in other high latitude temperate and boreal systems has similarly detected declines among species associated with northern systems. Niemi et al. [41], working in national forests of the western Great Lakes region, observed a significant decline in yellow-bellied flycatcher counts, and Ralston et al. [42] identified declines for olive-sided and yellow-bellied flycatcher as well as Canada jay in a combined analysis of bird monitoring data throughout the Northeastern and Midwestern US. Virkkala and Rajasärkkä [43] documented declines of species associated with mires and wetlands in boreal protected areas in Finland, and Laaksonen and Lehikoinen [44] also observed patterns of decline among northern birds in long-term Finish bird surveys. Northern birds have also demonstrated greater decline and/or range contraction in comparison to southern birds in boreal Sweden [45]. Feeding strategy, and specifically flycatching, was the only factor in addition to the boreal guild which explained significant variability among occupancy trend parameters for birds in our system. Decline among the flycatching and boreal guilds is most likely conflated in our dataset because 2 of the 4 flycatchers for which trends were calculated are boreal species (yellow-bellied and olive-sided flycatcher). Three additional flycatchers have been detected in our study locations including alder flycatcher (*Empidonax alnorum*), which we found to have a positive trend, least flycatcher (*Empidonax minimus*), which had a negative trend, and great-crested flycatcher (*Myiarchus crinitus*), for which insufficient data precluded trend assessment. The decline of aerial insectivores has been highlighted in several regions [46,47], and agricultural intensification [48], neonicotinoid insecticides [49], and climate change [50] have all been postulated as possible causes. The majority of our study site locations are located on permanently protected New York State Forest Preserve [51] lands in a region with little agriculture, and so it is more likely that climate factors would be behind observed declines of flycatchers in our study sites, though this does not preclude agriculture and pesticide effects on other aerial insectivore species (e.g., swallows) on private lands within the Adirondack Park.

### Question 2: Site-level community change

We explored changes in avian communities in boreal wetlands by modeling changes in relative species richness within each of our study locations independently. We found that the process of community change was not uniform across sites and that different factors appear to operate at different locations in structuring community change. Broadly, however, it appears that habitat affinity plays an important role in shaping which birds are colonizing and which are leaving these habitats.

Results suggest that conifer-associated species and commensal/generalist species are most likely to be increasing as a proportion of the bird community, while open land birds are more transient in low elevation boreal wetlands. These patterns may reflect broad-scale abundance changes documented in other studies. Both commensal species and forest birds are among the species with the most robust populations and least conservation concern based on North American population trends [52]. Forest birds have been shown to be increasing on state [53], regional [41] and continental scales, possibly as a result of forest growth following agricultural abandonment in the 20^th^ century [54,55]. Increases in generalist or human-adapted species have also been noted in several studies [55,56,57], with increasing simplification or homogenization of bird communities attributed to a variety of factors including urbanization [58], climate change [57], and peatland tree encroachment induced by land uses such as logging and agriculture [59]. The observed high extinction rates for birds of open lands may be a reflection that these habitats are less suitable for this guild overall. Open land birds constituted birds of both open woodland habitats and grassland landscapes with scattered trees such as farmlands and orchards. They may find suitable resources and/or lower rates of nest predation in the more open boreal wetland habitat types but may not constitute a major proportion of the bird community. Observed increases in richness of commensal species may not bode well for the more rare and specialized species at the center of our monitoring efforts. Although peatlands in general [17], as well as those here, are characterized by relatively low human impact in comparison with other habitat types, we have already found boreal specialist species to be sensitive to human footprint and more likely to abandon sites with higher degrees of human impact [14]. Biotic homogenization, as a result of the decline of specialists and rise of widespread generalists, is an increasingly recognized global phenomenon [60].

Migration strategy, nesting location, and southern vs northern distribution were also important characteristics linked to long-term changes in relative species richness in boreal wetlands. Because they are present on territories year round, resident birds are often theorized to be less sensitive to and more capable of tracking changing conditions over time [61], and increasing abundance and/or occupancy of resident birds relative to long- and short-distance migrants has been documented in several studies [415,61]. Though we found that northern species were still more likely to colonize and persist within these northern habitats, variable rates of colonization of southern birds across sites may indicate an increasing representation of southern species as temperatures warm, which may alter competitive dynamics in these habitats [8].

In concert, our findings with respect to the first two questions point toward potential long-term structural change. Though the majority of individual species were determined to have relatively stable occupancy trends (Question 1), with respect to changes in species richness in these communities, most guilds exhibited higher extinction rates than colonization rates (Question 2), suggesting potential overall declines in species richness over time. It is possible that, although most individual bird species are not exhibiting large changes in occupancy, some of the more specialized members of the community are declining (e.g., boreal or forest interior specialists) and these communities as a whole are shifting toward a smaller number of more common species.

### Question 3: Boreal decline

We found that extinction rates of boreal birds were lowest in sites with the largest amounts of Northern Peatland and lowest amounts of Boreal Upland Forest. Among our study sites, high amounts of Northern Peatland are associated with large wetland sites; some of the largest boreal wetlands in our system are those dominated by Northern Peatland habitat types such as Boreal Laurentian Bog and Boreal Laurentian-Acadian Acidic Basin Fen [22]. They are also characterized by generally lower amounts of human impact than the more forested boreal habitat types. In addition to the larger size and higher intactness of these habitats, they may be characterized by lower densities of red squirrels in comparison to Boreal Upland Forest habitat types. Red squirrels are major nest predators in northern forests [62] and, in comparison to forested conifer habitats, open bog may represent suboptimal habitat used by squirrels only during natal dispersal [63].

We also found that sites with low extinction rates for boreal birds were characterized by lower deviation in winter precipitation from normal conditions. Separate analyses of these data have found that boreal birds are sensitive to current temperature and precipitation patterns and that winter precipitation characteristics influenced the persistence of resident boreal bird species including black-backed woodpecker, boreal chickadee, and Canada jay [15]. Variability in winter precipitation may be detrimental to Canada jay, in particular, because its nesting period can begin as early as February and coincides with harsh winter conditions [64]. It is therefore possible that resident species may be better able to persist in locations where the climate is relatively more stable and similar to long-term conditions, relative to migratory species. We did not find strong support for the influence of temperature stability on declining species richness of boreal birds. Precipitation is, in general, more variable than temperature among our study sites however. This is especially true with respect to the extremes of precipitation which occurred at variable and inconsistent times of year during the course of our study. Modeling historical, current, and future climate in the Adirondacks is severely impeded by the complex topography of the region and the scarcity of weather stations [65,66]. We used available data from the most recent normals (1981–2010) because they provide the best information for directly assessing the deviation of current from long-term conditions, but it is possible that variability driven by local factors has obscured recent trends [65]. Nevertheless, sites with low extinction rates for boreal birds appeared to have more stable winter precipitation patterns. Low lying open peatlands are subject to cold air drainage [67]. Such cold-air pools may help maintain colder local climates and contribute toward the value of these locations as climate change refugia [19].

It is important to understand the degree to which changing avian species composition overall may also be contributing to the loss of boreal birds. We found sites with high extinction rates for the boreal bird guild had significantly higher colonization rates of both southern and commensal species than did sites with low or medium extinction rates. Increases among both groups represent possible responses to climate change and additional pressures on boreal birds in the form of competition for resources. Climate change may explain increasing southern bird occurrence [1] and is similarly implicated in the rise of generalist, and associated decline of specialist species, especially in concert with land use change [68]. There is overlap among the two guilds, with most of the commensal species in our study also being southern species, making it difficult to distinguish the potential effects of one versus the other on boreal bird dynamics. Nonetheless, climate and land use change (i.e. habitat loss and alteration) are likely to bring more generalist/human-adapted species into boreal habitats and these species, better able to exploit a variety of habitats and food sources, may outcompete the rarer boreal-adapted species [14,69,70].

## Conclusions

Our analysis revealed that (1) boreal species may be exhibiting the largest changes in occupancy among our study locations as compared to the larger avian community, (2) processes of community change are not uniform across sites but habitat structure may play an important role in driving observed changes, and (3) particular characteristics may allow some locations (e.g., large open peatlands) to serve as refugia for boreal species in the context of climate change. The largest open peatlands in the Adirondacks are generally well protected, but the network of smaller, more isolated boreal habitats exists among a variety of ownerships and land use types. Because boreal habitats in the Adirondacks are islands within a landscape dominated by temperate forest types, and because boreal birds within them may behave as metapopulations [14], protection of the variety of sizes and types of boreal habitats throughout the park is important to maintaining overall connectivity for this habitat type, and can benefit from wise use practices. Those which optimize water management (i.e., reduce drainage) may be most beneficial in combatting degradation and conserving biodiversity [17]. In the Adirondack Park, management practices relevant to timber harvesting, such as use of protective buffers on surrounding upland habitats, limiting management practices to thinning or other partial harvests rather than clearcuts, and restricting harvest to winter time may be most important. In addition, regulation of recreational access and use of boreal habitats is also highly relevant in the Adirondack Park. The placement of trails and roads is also an important component of maintaining healthy boreal habitat. By placing trails and roads that may be accessed by heavy machinery such as ATVs at least 50m from boreal wetlands, and avoiding trails through peatland habitat, managers can improve the resilience of these habitats to climate-driven changes. In popular destinations, raised boardwalks can help protect sensitive vegetation. Protection of uplands surrounding peatland areas is also critical; maintaining a minimal zone of 100m undisturbed by development or agriculture will benefit many species [71]. These and other wise use practices may be critical to helping to protect boreal bird refugia.

## Supporting information

S1 File Species characteristics and calculated occupancy trend parameters (λ). (XLSX)

S1 Table Models used to predict changes in species richness. (DOCX)

S2 Table Influence of species characteristics on changes in species richness. (DOCX)
