# Supplementary material for: Temporal changes in avian community composition in lowland conifer habitats at the southern edge of the boreal zone in the Adirondack Park, NY
Source: PLoS One. 2019 Aug 19;14(8):e0220927. doi: 10.1371/journal.pone.0220927 (PMC6699670; doi:10.1371/journal.pone.0220927)
Supplement: S1 Table — (DOCX) [file pone.0220927.s002.docx]

S1 Table. Models and species characteristics used to predict changes in relative species richness among bird communities in boreal wetlands in the Adirondack Park, NY, 2007 – 2016.

| Colonization Models | Extinction Models | Species Characteristic |
| --- | --- | --- |
| ψ (.), γ(boreal), ε(.) | ψ (.), γ(.), ε(boreal) | Boreal Distribution |
| ψ (.), γ(southern), ε(.) | ψ (.), γ(.), ε(southern) | Southern Distribution |
| ψ (.), γ(feeding), ε(.) | ψ (.), γ(.), ε(feeding) | Feeding Strategy |
| ψ (.), γ(foraging), ε(.) | ψ (.), γ(.), ε(foraging) | Foraging Method |
| ψ (.), γ(nesting), ε(.) | ψ (.), γ(.), ε(nesting) | Nesting Location |
| ψ (.), γ(habitat), ε(.) | ψ (.), γ(.), ε(habitat) | Habitat Affinity |
| ψ (.), γ(migration), ε(.) | ψ (.), γ(.), ε(migration) | Migratory Strategy |
| ψ (.), γ(winter), ε(.) | ψ (.), γ(.), ε(winter) | Winter Geography |
| ψ (.), γ(single), ε(.) | ψ (.), γ(.), ε(single) | Number of Broods |
| ψ (.), γ(clutch), ε(.) | ψ (.), γ(.), ε(clutch) | Mean Clutch Size |
| ψ (.), γ(weight), ε(.) | ψ (.), γ(.), ε(weight) | Mean Weight |
| ψ (.), γ(utmy), ε(.) | ψ (.), γ(.), ε(utmy) | Mean Latitude of NY Distribution |
| ψ (.), γ(nypop), ε(.) | ψ (.), γ(.), ε(nypop) | NY Population |
| ψ (.), γ(arrival), ε(.) | ψ (.), γ(.), ε(arrival) | Mean Arrival Date |
