# Supplementary material for: Temporal changes in avian community composition in lowland conifer habitats at the southern edge of the boreal zone in the Adirondack Park, NY
Source: PLoS One. 2019 Aug 19;14(8):e0220927. doi: 10.1371/journal.pone.0220927 (PMC6699670; doi:10.1371/journal.pone.0220927)
Supplement: S2 Table — (DOCX) [file pone.0220927.s003.docx]

S2 Table. Influence of 14 species characteristics on colonization (ϒ) and extinction (ε) rates within avian communities in boreal wetland habitats in the Adirondack Park, NY, 2007 – 2016. Top Models indicates the # of models with ΔAIC ≤ 2.0 that included each characteristic.

| Characteristic | Guild | ϒ | Top Models | ε | Top Models |
| --- | --- | --- | --- | --- | --- |
| Distribution | Boreal (Y) | 0.09 | 11 | 0.33 | 9 |
|  | Boreal (N) | 0.12 |  | 0.20 |  |
| Distribution | Southern (Y) | 0.08 | 21 | 0.31 | 12 |
|  | Southern (N) | 0.16 |  | 0.15 |  |
| Feeding Strategy | Insectivore | 0.11 | 7 | 0.18 | 1 |
|  | Omnivore | 0.13 |  | 0.20 |  |
| Foraging Method | Bark Forager | 0.14 | 4 | 0.33 | 6 |
|  | Flycatcher | 0.06 |  | 0.27 |  |
|  | Foliage Gleaner | 0.11 |  | 0.20 |  |
|  | Ground Gleaner | 0.14 |  | 0.17 |  |
| Nesting Location | Cavity | 0.10 | 8 | 0.34 | 22 |
|  | Ground | 0.14 |  | 0.09 |  |
|  | Shrub | 0.07 |  | 0.39 |  |
|  | Tree | 0.12 |  | 0.23 |  |
| Habitat Affinity | Commensal | 0.16 | 19 | 0.19 | 15 |
|  | Conifer Forest | 0.20 |  | 0.18 |  |
|  | Forest Interior | 0.14 |  | 0.33 |  |
|  | Forest General | 0.13 |  | 0.21 |  |
|  | Open | 0.05 |  | 0.55 |  |
|  | Scrub/Marsh | 0.13 |  | 0.19 |  |
| Migratory Strategy | Long Distance | 0.08 | 16 | 0.25 | 4 |
|  | Short Distance | 0.14 |  | 0.16 |  |
|  | Resident | 0.14 |  | 0.27 |  |
| Winter Geography | Caribbean | 0.08 | 0 | 0.18 | 2 |
|  | Central/South America | 0.10 |  | 0.20 |  |
|  | Resident | 0.11 |  | 0.43 |  |
|  | US/Canada | 0.14 |  | 0.15 |  |
|  | Widespread | 0.08 |  | 0.23 |  |
| Number of Broods | Single | 0.09 | 11 | 0.25 | 4 |
|  | Multiple | 0.12 |  | 0.16 |  |
| Clutch Size | NA | Mixed | 5 | Mixed | 3 |
| Body Weight | NA | Mixed | 12 | Positive | 10 |
| NY population | NA | Positive | 15 | Mixed | 8 |
| NY latitude | NA | Mixed | 7 | Mixed | 3 |
| Arrival date | NA | Negative | 4 | Negative | 1 |
